# Dance Fitness Classes Improve the Health-Related Quality of Life in Sedentary Women

**DOI:** 10.3390/ijerph17113771

**Published:** 2020-05-26

**Authors:** Yaira Barranco-Ruiz, Susana Paz-Viteri, Emilio Villa-González

**Affiliations:** 1 Department of Physical Education and Sports, PROFITH “PROmoting FITness and Health through Physical Activity” Research Group, Sport and Health University Research Institute (iMUDS), Faculty of Education and Sport Sciences, University of Granada, 52071 Melilla, Spain; evilla@ugr.es; 2 Pedagogy School of Physical Activity and Sports, Faculty of Education Sciences, National University of Chimborazo, Riobamba 060150, Ecuador; spaz@unach.edu.ec

**Keywords:** group fitness classes, sedentarism, physical activity, dance exercise

## Abstract

*Introduction*: This study aims to analyze the effect of two dance-focused and choreographic fitness classes on Health-Related Quality of Life (HRQoL) in sedentary worker women. *Methods*: 65 sedentary middle-aged worker women (38 ± 7.3 years old) completed a 16-week intervention randomly assigned to: (1) dance fitness group based on Zumba Fitness classes (DF group, *n* = 25)], (2) dance fitness + functional strength training group (DFFT group, *n* = 20), and (3) control group (*n* = 20). HRQoL was assessed by the 36-Item Short-Form Health-Survey (SF-36), which evaluates 8 dimensions of health [General Health (GH), Physical Functioning (PF), Social Functioning (SF), Physical Role (PR), Emotional Role (ER), Bodily Pain (BP), Vitality (V), and Mental Health (MH)] scored from 0 (worst) to 100 (best health status). *Results*: The control group statistically differed from both exercise groups in PF and PR, and from the DF group in SF and MH showing a lower score. No statistical differences were observed between exercise groups post-intervention, except in V. DF group showed increases in GH, PF, SF, V, PR, and MH post-intervention. *Conclusions*: A 16-week dance fitness intervention based on Zumba Fitness classes generates notable improvements in a wide range of HRQoL dimensions in sedentary middle-aged worker women, especially in V, PR and MH dimensions.

## 1. Introduction

Health-Related Quality of Life (HRQoL) is defined as a multidimensional concept mainly based on a person’s subjective judgment of physical, functional, emotional, and social well-being [1]. It is considered an important public health research topic and could contribute to the detection of health problems beyond medical controls [2,3]. HRQoL has been widely studied in populations with some chronic or specific disease or pathologies [4], such as cancer [5], diabetes mellitus [6], heart disease [7], obesity [8], hypertension [9], lupus [10], generalized chronic pain [11] among others. However, HRQoL in apparently healthy adults has been little studied [12], despite the fact that they represent a high percentage of the population who also demand health services. Their stressed and mainly sedentary daily work activity makes this population also a group at risk. Therefore, knowing the perceived well-being and health of apparently healthy adults could help to prevent the possible economic, social, and public health effects derived from the common diseases in working adults, consequently becoming an important research field [13]. 

Furthermore, higher levels of physical activity (PA) are associated with better HRQoL [14,15]. Practicing PA in leisure time, in addition to increasing fitness levels and improving body composition as a protective tool for health, has been shown to improve several important areas of quality of life such as sociability and emotional and mental health [2,16]. However, the trend in the prevalence of physical inactivity continues to increase, especially in women from Latin America and the Caribbean [17]. Additionally, work-related PA has decreased over the last decades with the increase in mainly sedentary task jobs, which in energy expenditure terms correspond with a reduction of more than 100 calories per day [18]. This has caused an increase in total sedentary time, concerning more directly women, who are usually less active than men (31%–7% versus 23.4%, respectively) [17]. These poorer rates of daily PA have extensive effects on women’s health and well-being [19], considering that insufficient physical activity is a leading risk factor for non-communicable diseases and can also negatively affect mental health and quality of life [20]. Also, feminine gender was associated with a poorer perception of HRQoL compared with the masculine [21]. Therefore, new approaches that address the gender gap in physical activity to improve the quality of life are needed [22]. In addition, the great challenge of increasing PA levels is preserving the adherence to a physical exercise intervention in adulthood, since adults get discouraged from including PA in their daily routine. In that regard, contact, and social support in physical activity interventions are crucial to ensure adherence [23]. 

In this sense, dance appears as a type of PA with several health benefits; for example, it could reduce depression and anxiety and increases quality of life, interpersonal and cognitive skills, as well as psycho-motor skills [24]. Dance has been described as a worldwide form of cultural expression, which creates an ideal atmosphere for practicing physical activity [25] with the integration and construction of cognitive processes, emotions, and the self’s identity through experience and awareness of the body in movement [26]. Since ancient times, dance has been considered a therapeutic and attractive exercise, especially for women [27]. However, little is known about the effects of the new modalities of the dance, such as fitness trends like the Zumba Fitness classes on HRQoL. Dance exercise intervention of any kind is comparable to and sometimes more effective than other types of structured exercise for improving several health dimensions [28]. In this regard, group fitness activities are a successful strategy to increase physical activity in adults with theoretical and empirical support [29]. Moreover, nowadays, group fitness activities also are considered one of the most popular physical activities among the female audience [30,31]. However, there are few studies that evaluate the effect of group-based dance exercise interventions with musical support on the quality of life in sedentary worker women. According to previous studies, interventions based on dance or choreography could show us an improvement in the HRQoL [32], even in short interventions [33]. Consequently, it is expected that group-based exercise interventions based on fitness trends that use dance and music as an essential feature will improve several HRQoL dimensions in sedentary middle-aged worker women. Therefore, the objective of this study was to analyze the effect of two exercise interventions based on dance-focused and choreographic group fitness classes on HRQoL in sedentary worker women.

## 2. Materials and Methods 

### 2.1. Ethics Approval and Consent to Participate

This study is part of the “For a healthy university project” approved by the National University of Chimborazo Research Committee (29-CI-2014-10-17-22). All procedures were performed in accordance with the Declaration of Helsinki for research on human subjects. All participants provided informed consent prior to participation. 

### 2.2. Participants

**Figure 1:**
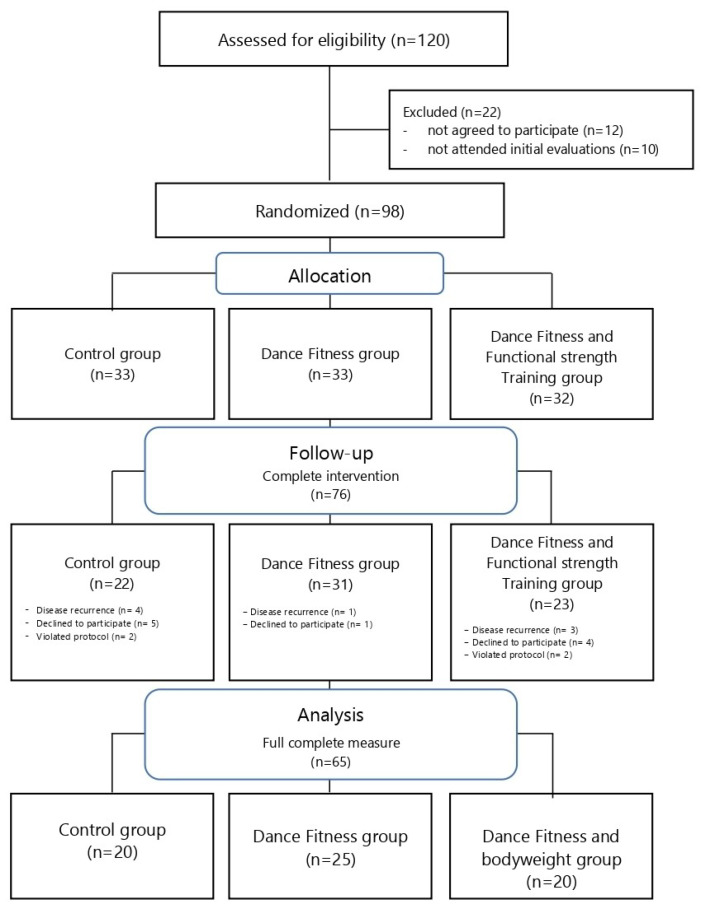
Flow diagram of the study.

Inclusion criteria were apparently healthy inactive adult women (from 25 to 50 years old, and less than 150 min of physical activity per week), and university workers with mainly sedentary tasks in her job occupation (sitting for more than 6 h a day). Women with a serious illness diagnosed, such as cancer, stroke, or severe muscular illness were excluded from the study. Participants were invited to the study by email requests from university corporative accounts (approx. 948 university women workers). A total of 150 interested women responded to the email invitation and 148 attended the initial study meeting. Finally, 120 participants accomplished the inclusion criteria and 108 agreed to participate in the study, but only 98 attended the initial evaluation. These 98 participants were randomly assigned to the study groups. The randomization process was done by a member of university staff who was not part of the research team. One academic staff extracted one by one ballots from a central box (each ballot belonged to a code assigned to a participant) and distributed it always from right to left into 3 boxes (each assigned to a study group). Neither this person nor the team of researchers knew which box was assigned to each study group or which code belonged to each participant. After the evaluation, 76 participants concluded the intervention period, and 65 were analyzed for this study since they fully completed the study measurement instrument (Figure 1).

Participants of this study were analyzed in their corresponding study group (one control group and 2 experimental groups). The habitual lifestyle without exercise intervention was followed in the control group (*n* = 20). The two experimental groups were based on exercise interventions 3 days per week outside working hours at 6.00 pm in the sport facilities of the university. One experimental group performed a dance fitness intervention based on group-based Zumba Fitness classes [dance fitness group (DF group, *n* = 25)], whereas the second experimental group simultaneously performed the Zumba fitness classes with the DF group, and then added 20 min of functional strength training with bodyweight [dance fitness and functional strength training group (DFFT group, *n* = 20)]. Additionally, along the intervention period, the three study groups received two nutritional education sessions with recommendations for how to adopt healthy nutrition habits. Moreover, the research team offered to the control group the possibility to perform the exercise intervention after the study.

### 2.3. Interventions

The dance fitness intervention was based on a Zumba Fitness program. Zumba Fitness classes were led by a professional Zumba instructor (ZIN member). The Zumba Fitness classes performed in this study followed the choreographies and the class structure of the official Zumba ZIN DVD (48 and 50). Generally, the classes consisted of approx. 60 min of activity that began with 5–10 min of warm-up with one or two Latin music tracks focused on joint mobility and dynamic flexibility, followed by the main part (40 to 45 min) with 6 to 8 tracks with the combination of different Latin rhythms, and concluding with 5 to 10 min for returning to calm by dynamic stretching and breathing movements through soft Latin rhyths such as bachata, kizomba, etc., or slow music.

The Functional Strength training consisted in performing whole-body functional bodyweight exercises with musical support. Five music tracks of 4 min each were chosen to train the following muscle groups: lower limbs, chest, upper limbs, abdomen, and lower back. The exercises were executed at different speeds according to the music tempo (tempo or double tempo). Examples of whole-body functional exercises are squats, lunges, push-up, crunches, triceps dips, bodyweight pulls, glute bridges, and low-back extensions. The intensity of the training sessions was controlled by the 0–10 Rating Perceived Exertion Borg Scale (RPE) [34]. A RPE session approach was performed before intervention beginning in order to explain the meaning and correct application of the Borg Scale to the participants. The intensity changes during the training sessions were indicated by the instructor in order to control that no one made strenuous efforts and maintained a moderate-to-vigorous- intensity physical activity (6 to 8 in the Borg Scale). At the end of each session, participants declared the average intensity of the session and the attendance.

### 2.4. Health-Related Quality of Life

Health-Related Quality of Life (HRQoL) was assessed by a cross-cultural adaptation of the Spanish version of the 36-Item Short-Form Health Survey (SF-36) [35]. The SF-36 questionnaire asseses the perceived degree of health. The questionnaire is composed of 36 items, where 35 of them analyze 8 dimensions of health: General Health (GH), Physical Functioning (PF), Social Functioning (SF), Physical Role (PR), Emotional Role (ER), Bodily Pain (BP), Vitality (V), and Mental Health (MH)), and 1 item analyzes the Declared Evolution of the Health. Each dimension has a number of items, and each item has a number of answer options (Table 1). For the analysis, the score was standardized for each item in a range from 0 to 100 (0 = worst; 100 = best health status). The average of score between the items of each dimension was used to evaluate the perceived health status for that dimension of the QoL. The test-retest correlation coefficients of reliability and internal consistency of the SF-36 questionnaire have been previously reported in the adult population with optimal ranges exceeding recommended standards (0.58–0.99 and Cronbach’s alpha of 0.78–0.96) [36].

### 2.5. Statistical Analysis

Data are expressed as mean ± standard deviation (SD), and mean differences (MD) and standard error of the mean differences (SEM). The normality of distribution was assessed with the Kolmogorov–Smirnov test. Due to the design of the study, the effects of the interventions and interaction between study factors (study groups and moments of assessment) were analyzed by a mixed factorial ANOVA. Paired comparisons within the groups and between groups were analyzed by the Bonferroni post hoc test using age, adherence and baseline values of each variable as covariables. The statistical software SPSS 26.0 of IBM SPSS (Armonk, NY, USA) was used for all the analyses, establishing the significance level at *p* < 0.05.

## 3. Results

A total of 65 participants (38.0 ± 7.3 years old) fully and correctly completed the SF-36 questionnaire. The average of attendance and perceived intensity during the intervention sessions were 79.7 ± 7.6 and 7.6 ± 0.8, respectively (DF group = 7.5 ± 0.8 and DFFT group = 7.7 ± 0.7).

### 3.1. Changes in HRQoL Dimensions from Baseline to Post-Intervention within the Groups

**Figure 2:**
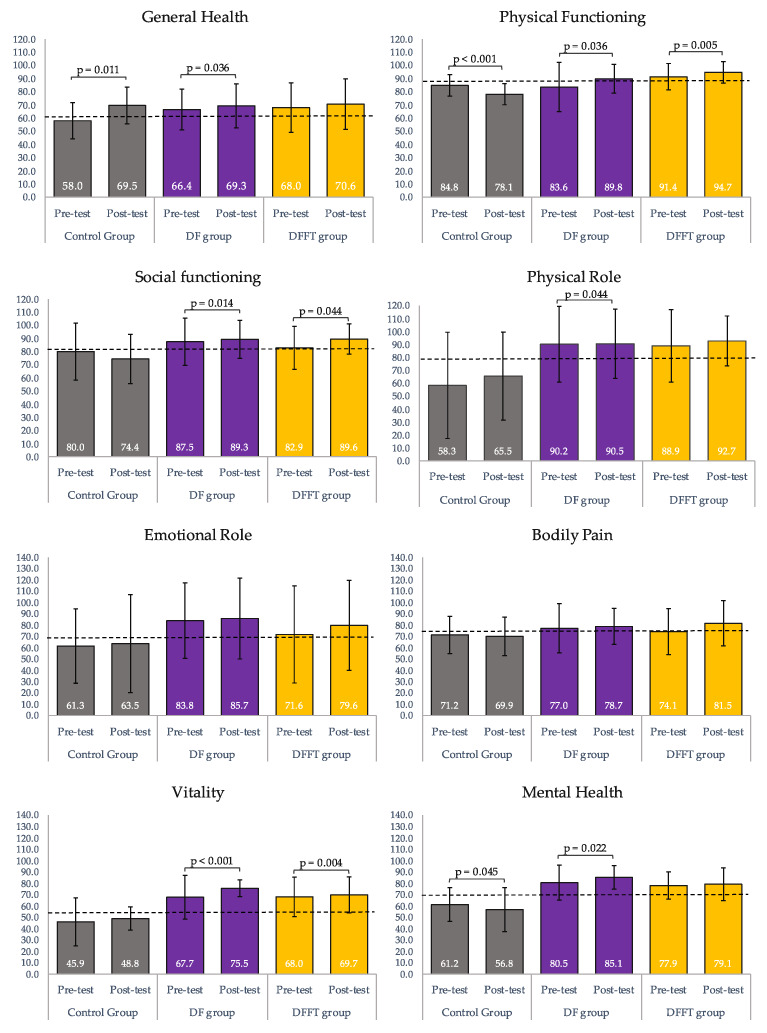
Changes in HRQoL dimensions from baseline to post-intervention within the study groups (mixed factorial ANOVA, Bonferroni post-hoc test for differences pre-post within the groups). DF group = Dance Fitness group; DFFT = Dance Fitness and functional strength training group; Data are expressed as mean (M) and standard deviation (SD) based on marginal means and the following baseline leves of reference represented by discontinued line: GH = 62.14, PF = 86.30, SF = 80.68, PR = 80.45, ER = 69.05, BP = 74.07, V = 56.8, MH = 70.07.

**Figure 3:**
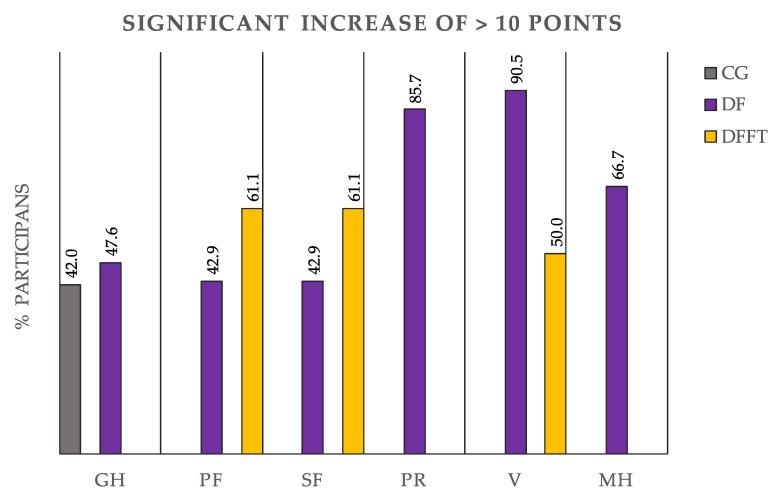
Percentage of participants with a significant score improvements of more than 10 points compared with the baseline. General Health (GH), Physical Functioning (PF), Social Functioning (SF), Physical Role (PR), Vitality (V), and Mental Health (MH). DF group = Dance Fitness group; DFFT = Dance Fitness and functional strength training group.

Changes in HRQoL dimensions from baseline to post-intervention within the groups are presented in Figure 2. In brief, six out of eight HRQoL dimensions significantly improved at the post-intervention in the DF group, whereas three of eight dimensions significantly improved in the DFFT group, as well as in DF group. The HRQoL mainly remained with no post-intervention changes for the control group, which presented detriments in some HRQoL dimensions. The general health dimension significantly improved in the DF group and Control group (MD = 7.7 ± 3.6, *p* = 0.036; MD = 9.6 ± 3.6, *p* = 0.011, respectively), whereas improvements without significant changes were observed in the DFFT group. Physical Functioning, Social Functioning and Vitality significantly improved in both exercise intervention groups (DF group: MD = 6.2 ± 1.8, *p* = 0.001, and DFFT group: MD = 5.7 ± 1.9, *p* = 0.005; DF group: MD = 8.8 ± 3.4, *p* = 0.014 and DFFT group: MD = 7.3 ± 3.5, *p* = 0.044; DF group: MD = 17.6 ± 2.4, *p* < 0.001; DFFT group: MD = 8.3 ± 2.7, *p* = 0.004, respectively). The DF group also presented a positive increase in Physical Role (MD = 13.9 ± 5.9, *p* = 0.022) and Mental Health (MD = 14.2 ± 3.4, *p* < 0.0001). The control group showed significant detriments in the Physical Functioning and Mental health dimensions (MD = 8.2 ± 1.7, *p* < 0.000 and MD = 8.2 ± 3.8, *p* = 0.033). The percentage of participants within the study groups with score improvements of more than 10 points is presented in Figure 3. The DF group has the greatest percentage of participants with significant increments of more than 10 points (high improvements). Concretely, the majority of the participants of the DF group presented high improvements in three of the six dimensions in which presented significant changes after the intervention: Vitality (90.5%), Physical Role (85.7%), and Mental Health (66.7%). Less than 50% of the participants reached high improvements compared with the baseline values in the HRQoL dimensions of General Health (47.6%), Physical functioning (42.9%) and Social Functioning (42.9%). Moreover, the DFFT group showed high improvements in 61.1% of participants for Physical and Social Functioning, as well as, in 50.0% of the participants for the Vitality dimension. The control group only presented significant-high improvements in the 42.0% of the participants for the General Health dimension.

### 3.2. Comparisons between Study Groups Post-Intervention

After intervention, the control group statistically differed from both exercise groups in Physical Functioning (Control group versus DF group: MD = −14.43 ± 2.48, *p* < 0.001; Control group versus DFFT group: MD = −13.85 ± 2.6, *p* < 0.001), and Physical Role (Control group versus DF group: MD = −12.94 ± 4.20, *p* = 0.010; Control group versus DFFT group: MD = −11.51 ± 4.380, *p* = 0.034) showing lower punctuation. Statistical differences were also observed between the control group and DF group in the Social Functioning and Mental Health dimensions, where the control group presented lower punctuation (MD = −12.95 ± 4.80, *p =* 0.029 and MD = −22.411 ± 5.34, *p* < 0.001, respectively). All study groups differed in Vitality dimension (Control group versus DF group: MD = −22.06 ± 3.74, *p* < 0.001; Control group versus DFFT group: MD = −12.78 ± 4.12, *p* = 0.009), and DFFT group versus DF group: MD = −22.41 ± 5.4, *p* < 0.001) where the DF group reached the highest score. No statistical differences were observed between groups post-intervention for General Health, Emotional Role, and Bodily Pain. 

## 4. Discussion

The main findings of the present study were that both exercise intervention groups significantly improved in several HRQoL dimensions at the post-intervention compared with the control group, for which the HRQoL dimensions mainly remained without changes post-intervention or even presented decreases. On the other hand, six out of the eight HRQoL dimensions significantly improved post-intervention in the DF group, whereas three of eight dimensions significantly improved in the DFFT group, having thus both groups improvements compared with the control group. 

To date, while dance intervention studies focused on health, quality of life, and well-being have increased in the elderly [37] adolescent [38,39], and clinical samples [40,41,42], the relationship between dance practice and HRQoL in non-clinical samples of women are scarce. The current work is one of the few studies testing the hypothesis that a dance exercise intervention based on Zumba fitness classes positively affects HRQoL sedentary middle-aged worker women. To our knowledge, only five studies have evaluated the effects of dance exercise intervention based on Zumba Fitness classes on HRQoL in apparently healthy women [33,43,44,45,46] with positive effects on most of the dimensions of HRQoL. In two of these studies, participants had special conditions, such as postmenopause and/or overweight women [45,46]. In the case of a study carried out in a sample of female college students (mean age 21 years old), an 8-week Zumba Fitness intervention (twice weekly) also improved the total sum of scores related to HRQoL measured by the WHOQoL questionnaire with a large magnitude effect (partial η2 = 0.45) [47]. In another study carried out in a randomized sample of fifty-three older women, most of the HRQoL measures using the SF-36 questionnaire (physical functioning, role-physical, bodily pain, vitality, social functioning, role emotional, physical dimension, and mental dimension) differed significantly from the control group after 3 months, but not after 6 months of a twice-weekly Zumba Fitness intervention [48]. Additionally, in the case of women with fibromyalgia a Zumba Fitness intervention only improved physical functioning [49]. Finally, two recent systematic reviews about the health benefits of Zumba fitness concluded that Zumba Fitness could be considered as an effective type of physical activity able to improve mainly aerobic capacity, as well as psychological and social aspects concerning the quality of life [50,51]. 

In the current study, most HRQoL dimensions (6/8) significantly improved post-intervention in the DF group, which performed a 16-week Zumba Fitness intervention (3 times per week/60 min per session). Although there are very few studies in the literature, our finding is in line with previous research that has shown QoL improvement after a Zumba Fitness intervention in women [33,43,44,45,46,47]. Our findings indicated that a Zumba Fitness intervention isolated (i.e., without an additional functional strength training) could generate improvements in the majority of the HRQoL dimensions, specifically in Physical Functioning, Social functioning, Vitality, Physical Role, Mental Health, and General Health. Similar results were observed in a previous study with only five weeks of intervention [33], where the same HRQoL dimensions have improved except for Physical Role; unlike the present study, in the study with 5 weeks of intervention, the emotional role experienced a great increase (+17 points). In contrast, only 2 dimensions of QoL (Physical Functioning and Emotional Role) were positively affected by a 12-week Zumba fitness intervention in sedentary overweight women [45]. Half of the quality of the life dimensions (4 out 8) improved positively in two studies despite differing considerably in the duration of the intervention with 8 weeks [43] and 6 months, respectively [44]. In the study with the longest intervention [44], significant increases in the Physical Functioning, Physical Role, Bodily Pain, and Social Functioning scores were observed, whereas the one with the shortest intervention period [43] improved General Health, Physical Functioning, Vitality and Emotional Role. However, in the extensive Zumba Fitness intervention study [44], the weekly frequency and the total of sessions were not declared, making difficult to compare results with the rest of similar studies. 

Most of the studies that analyzed HRQoL after a Zumba Fitness intervention found improvements between 5 to 10 points compared with the baseline scores in several dimensions of QoL [43,45,47]. In our study, the score increments were very similar to Domene et al.’s study [43] although only half of the weeks were performed compared with our study (8 versus 16 weeks). Our results showed increments from 6 to 17 points, reaching the highest mean increment in the Vitality dimension, similar to Domene et al., where the Vitality dimension reached also the highest improvement of 15.5 points compared with the baseline value. Moreover, according to previous findings found by our research group [33], a high percentage of participants experienced improvement above 10 points compared with baseline. Concretely in the present study, Vitality, Physical Role, and Mental health dimensions of HRQoL increased more than 10 points after the intervention in 90.47%, 85.71%, and 66.67% of participants, respectively, showing higher prevalence than in our previous study with 5 weeks of the intervention [33], where improvements by 10 points or more were observed in 63.4% of the participants for General Health and 58.3% of the participants for Social Functioning. Maybe, a more extensive intervention period or other confounding factors could explain greater improvements. Regarding DFFT intervention effect on HRQoL, no similar studies were found to compare our results. However, a recent review concluded that combining endurance and resistance training in the same training session (i.e., concurrent training) similar to the DFFT intervention in our study is an effective method for enhancing overall fitness as well as improving quality of life in this population, i.e., adult women [52]. In order to explain why the DFFT group presented statistical improvements in fewer dimensions than in DF group, we hypnotized that maybe the duration of the sessions (additional 20 min of training) or the type of class (i.e., more focused on improving strength and not dancing) could lead to a greater lack of motivation for a sample of sedentary women with low training experience. Consequently, QoL perception could have been not so positively affected. Maybe an easy and enjoyable choreographic dance class could be much better in this sample, being a sufficient stimulus that could improve their quality of life and other associated variables. Regarding the improvement in the General Health dimension in the Control Group, this could be explained by the fact that this group showed the lowest value in this dimension at baseline, and maybe following the given recommendations for healthy nutrition habits could have changed their perception of a healthier general status compared with baseline. In relation to the detriments after the intervention period in the control group, our results are in line with the previous studies, which included a control group, probably because in our study, although the women were apparently healthy, they hypothetically spent a large number of hours sitting at their workplace, generating detriments in their quality of life [53]. 

Concerning the comparison of the study groups after the intervention, no statistical differences were observed between the exercise intervention groups except in the vitality dimension where the DF group presented a significantly higher score compared with the DFFT. However, the control group differed from both exercise intervention groups, showing lower scores in physical functioning, physical role, and mental health. Additionally, the control group presented statistically lower values in social functioning compared with the DF group. Similar results were obtained in the Notarnicola et al. [44] study, where the Zumba Fitness intervention group statistically differed from the control group for Physical Functioning, Physical Role, Social Functioning, and Bodily Pain on a sample with similar age mean. In the present study, no differences were observed for Bodily Pain, however, mean differences in the present study were higher than in Notarnicola et al. for several dimensions such as Physical functioning, Physical Role, and Social Functioning. Also, the present study showed very similar mean differences to those presented in Domene et al. [43] for the physical functioning and physical role dimensions when Zumba Fitness and the control group were compared. Concretely, the mean differences after intervention between the Zumba Fitness group and the control group were around 13 and 12 points in both studies, respectively. The mental health score (emotional well-being) also differed between the Zumba Fitness group and the control group; however, greater mean differences were found in our study (+ 22.41 points) compared with the Domene et al. study (+ 9.8 points). 

Physical Functioning, Social Functioning and Emotional Role dimensions are the HRQoL dimensions that tend to show consistent improvements in most Zumba Fitness intervention studies. In line with this, in the present study Physical Functioning and Social Functioning were also improved in both dance exercise intervention groups. Physical Functioning and Physical Role are related to the extent to which health limits physical activities, work, and other daily activities, which are strongly associated with the physical fitness level. In this sense, this improvement could be an expected result since it has been demonstrated that Zumba Fitness interventions improve physical fitness levels, especially cardiorespiratory fitness [50,51,54]. Additionally, the social functioning dimension referring to physical or emotional health problems that interfere with normal social life also improved in both exercise dance intervention groups. It could be related to the positive social climate that is created when people exercise in a group [55]. People who practice group fitness classes feel that they are like an authentic group, which increases social and cohesion feelings and adherence to exercise [56]. Moreover, the groupness has a considerable effect on exertion, enjoyment, and affective perception during the participation in group fitness classes. The larger the groupness is, the greater exertion, enjoyment, and affective perceptions have been reported in participants of group fitness classes [56]. This fact could guarantee adherence to physical activity and could explain the perceived improvements related to Vitality showed in our study. Finally, although no improvements were observed for the emotional role, the participants manifested a notable improvement in the Mental Health dimension for both dance-fitness exercise groups. The scientific evidence supports that group musical activities with synchronized exertive movements like in dance group fitness classes have effects on mood and emotion causing affiliative sentiments and behaviors, which generate a positive effect on social bonding and well-being [57,58]. These phenomena are all strongly associated with the release of endorphins when listening to music, which is enhanced when the movement is involved, even more, when the movement is synchronized in a group [59]. For all these reasons, dance fitness group classes like Zumba Fitness classes should be considered an excellent strategy for improving HRQoL in adult women, beyond the known effects on physical and metabolic parameters [50]. Future research should explore the psychological benefits of structured exercise dance programs like Zumba Fitness in comparison to other structured exercise programs without choreographic and musical support.

Our study has both limitations and strengths. First, since the effects of exercise training on HRQoL are known to be affected by age and other confounding factors [60] and taking into account that our study sample was small and limited to sedentary adult women, these findings may not be generalizable to other populations with different characteristics. Second, the SF-36 is a self-reported instrument, however, the test-retest correlation coefficients of reliability and internal consistency of the questionnaire showed optimal ranges exceeding recommended standards (0.58–0.99 and Cronbach’s alpha of 0.78–0.96). Although it is not the objective of this study, it would has been interesting to perform a follow-up measure to analyze the self-efficacy of this type of exercise intervention on the physical activity levels of these women and on their HRQoL. Additionally, to our knowledge, this is the first study to examine the effect of two dance-focused and choreographic fitness classes (i.e., DF and DFFT) on HRQoL in sedentary middle-aged worker women without any clinical condition.

## 5. Conclusions

A 16-week group-based dance fitness intervention based on Zumba Fitness classes (3 times per week, 60 min per session) generates notable improvements in a wide range of HRQoL dimensions in sedentary middle-aged women, especially in vitality, physical role and mental health dimensions. Interventions such as dance-focused and choreographic structured group fitness classes like Zumba Fitness could be an alternative exercise modality that can help reduce the health risks associated with sedentary behaviors and improve the quality of life in sedentary adult women.

## Figures and Tables

**Table 1:** Short-Form Health-Survey Questionnaire (SF-36): dimensions and its meaning.

Quality of Life Dimensions	Number of Items	Number of Answer Options	Summary of Content and Meaning
General Health (GH)	5	5	Personal valuation of health including current health, future health prospects, and resistance to illness.
Physical Functioning (PF)	10	3	Extent to which health limits physical activities such as self-care, walking, climbing stairs, bending, picking up or carrying weights, and moderate and intense efforts.
Social Functioning (SF)	2	5	Extent to which physical or emotional health problems interfere with normal social life.
Physical Role (PR)	4	2	Extent to which physical health interferes with work and other daily activities, including less than desired performance, limitation in the type of activities performed, or difficulty in performing activities.
Emotional Role (ER)	3	2	Degree to which emotional problems interfere with work or other daily activities, including reduced time spent on those activities, less than desired performance, and decreased care while working.
Bodily Pain (BP)	2	5	The intensity of pain and its effect on regular work, both outside the home and at home.
Vitality (V)	4	6	Feeling of energy and vitality, compared to the feeling of exhaustion.
Mental Health (MH)	5	6	General mental health, including depression, anxiety, behavioral and emotional control, and the overall positive effect.
Declared evolution of health (DEH)	1	5	Current health assessment compared to a year ago.
